# Nephrotoxicity Evaluation of Indium Phosphide Quantum Dots with Different Surface Modifications in BALB/c Mice

**DOI:** 10.3390/ijms21197137

**Published:** 2020-09-27

**Authors:** Li Li, Tingting Chen, Zhiwen Yang, Yajing Chen, Dongmeng Liu, Huiyu Xiao, Maixian Liu, Kan Liu, Jiangyao Xu, Shikang Liu, Xiaomei Wang, Guimiao Lin, Gaixia Xu

**Affiliations:** 1 Base for International Science and Technology Cooperation, Carson Cancer Stem Cell Vaccines R&D Center, Shenzhen Key Lab of Synthetic Biology, Department of Physiology, School of Basic Medical Sciences, Shenzhen University, Shenzhen 518055, China; lili2017@szu.edu.cn (L.L.); jkf_ctt@szu.edu.cn (T.C.); yangzw@mail.sustech.edu.cn (Z.Y.); yajing9612@outlook.com (Y.C.); 1800243043@email.szu.edu.cn (D.L.); haha1250@sina.com (K.L.); 1800243016@email.szu.edu.cn (J.X.); 1800243019@email.szu.edu.cn (S.L.); xmwang@szu.edu.cn (X.W.); 2 Dongguan Key Laboratory of Environmental Medicine, School of Public Health, Guangdong Medical University, Dongguan 523808, China; 3 Shenzhen Institute for Drug Control, Shenzhen 518000, China; 2172281525@email.szu.edu.cn; 4 Guangdong Key Laboratory for Biomedical Measurements and Ultrasound Imaging, School of Biomedical Engineering, Health Science Center, Shenzhen University, Shenzhen 518055, China; maxliu@szu.edu.cn

**Keywords:** InP/ZnS quantum dots, surface chemistry, renal toxicity, oxidative stress, apoptosis

## Abstract

InP QDs have shown a great potential as cadmium-free QDs alternatives in biomedical applications. It is essential to understand the biological fate and toxicity of InP QDs. In this study, we investigated the in vivo renal toxicity of InP/ZnS QDs terminated with different functional groups—hydroxyl (hQDs), amino (aQDs) and carboxyl (cQDs). After a single intravenous injection into BALB/c mice, blood biochemistry, QDs distribution, histopathology, inflammatory response, oxidative stress and apoptosis genes were evaluated at different predetermined times. The results showed fluorescent signals from QDs could be detected in kidneys during the observation period. No obvious changes were observed in histopathological detection or biochemistry parameters. Inflammatory response and oxidative stress were found in the renal tissues of mice exposed to the three kinds of QDs. A significant increase of KIM-1 expression was observed in hQDs and aQDs groups, suggesting hQDs and aQDs could cause renal involvement. Apoptosis-related genes (Bax, Caspase 3, 7 and 9) were up-regulated in hQDs and aQDs groups. The above results suggested InP/ZnS QDs with different surface chemical properties would cause different biological behaviors and molecular actions in vivo. The surface chemical properties of QDs should be fully considered in the design of InP/ZnS QDs for biomedical applications.

## 1. Introduction

The integration of emerging nanotechnology into various biomolecules has aroused great interest in the fields of biology and medicine. Quantum dots (QDs), composed of group II–IV or III–V elements, have been extensively used in a wide range of biomedical applications and shown great potential in imaging, therapeutic functions and biosensors [1]. Compared with conventional fluorescent materials, QDs have the characteristics of high quantum yield, light bleaching resistance, a wide absorption spectrum, and a narrow and symmetrical emission spectrum [2]. The outer layer of QDs can be functionalized by different materials for different purpose. With these advantages, QDs have broad application prospects in biomedicine. For example, functionalized QDs can be used as drug carriers for high-precision delivery, and as fluorescent probes for specific organ or tumor imaging in vivo [3,4]. Therefore, the biological toxicity caused by QDs has also attracted the attention of researchers, and many toxicological studies on QDs have been carried out for this purpose.

Cadmium (Cd)-based QDs and tellurium (Te)-based QDs were first synthesized and have been placed in a very broad application prospect. Since these QDs contain toxic heavy metal elements (e.g., Cd, Te, lead (Pb), mercury (As) and hydrargyrum (Hg)), their toxicity in vivo and in vitro has attracted wide attention. The reported harmful effects of QDs include altering cell growth, changing gene expression, DNA damage and inducing cell apoptosis from humans cells, mice, zebrafish and even microorganisms [5]. After intravenous injection, QDs tend to be removed from blood through the reticular endothelial system of the liver and spleen, accumulate in the liver and kidney of mice, and cause potential damage [6,7]. Chen et al. [8] revealed that CdTe QDs could aggregate in human hepatocellular carcinoma cells (HepG2), human kidney cells 2 (HK-2) and Madin–Darby canine kidney (MDCK) cells, and cause significant toxicity in all the three cell lines. Zhao et al. [9] reported that Cd-QDs could enter cells in a time- and dose-dependent manner, interfere with redox balance in vivo, and have adverse effects on the kidney. Numerous previous studies have reported that the biological toxicity of heavy metal-based QDs is mainly attributed to the release of toxic ions such as cadmium ions from the core. On the other hand, the intrinsic toxicity of Cd-based QDs has very limited potential for real-life bioimaging and diagnostics in vivo, even without considering the potential impact of Cd-QDs on the environment [10,11]. In light of this, eco-friendly QDs have been developed and attracted widespread attention in the past decade.

Among all the alternative semiconductor materials, indium phosphide (InP) QDs were considered as one of the most promising candidate materials [12]. The photoluminescence (PL) wavelength of InP QDs can be tuned from the blue to the near-infrared (NIR) region by varying the particle size, which is similar to that of CdSe QDs. The encapsulation of an InP core with a ZnS shell can passivate surface defects, restrict excitons, prevent core oxidation and significantly increase the PL quantum yield of InP/ZnS core/shell QDs [13,14]. With the advantages of high sensitivity and low toxicity, high-performance InP QDs have been used as fluorescent probes for cell imaging, cell diagnosis and even antibiotic potentiators [15,16]. In the process of production, application or environmental exposure, QDs enter the organism through respiration, skin contact, consumption, intravenous injection and other routes. In addition, drug therapy and biological monitoring are also potential sources of QDs exposure. There are notable differences in the absorption, transfer, accumulation, degradation and exclusion of different QDs in organisms. These differences mainly depend on the physical and chemical properties of QDs, such as particle size, surface charge, surface chemical properties, etc. Several studies have reported that the biological toxicity of InP/ZnS QDs was lower than that of Cd-based QDs [17,18]. Brunetti et al. indicated that water-soluble InP/ZnS QDs were less toxic than CdSe/ZnS QDs to human A549 cells, SH-SY5Y cells and Drosophila, and they concluded that InP QDs were safer alternative nanomaterials to Cd-based QDs for biological applications [19]. Chen et al. found that In-based QDs could not change the morphology of Hydra polyps even at the highest experimental concentration, while Cd-based QDs displayed evident morphological changes, leading to death at 72 h [20]. However, some studies have reported that InP QDs have obvious toxicity. Huang et al. found that InP/ZnS QDs could strongly bind to subdomain IIA of human serum albumin (HSA) and cause more global and local conformational changes to HSA HAS than CdSe/ZnS QDs, which illustrated that InP/ZnS have higher potential toxicity [21]. Hence, it is still necessary to systematically study the toxicity of InP QDs in vivo and in vitro, and to further understand the mechanism of InP QDs.

The kidney is an important organ for eliminating metabolites, wastes and toxic materials. The nephron, consisting of renal corpuscles and tubule systems, is the structural and functional unit of kidney. Nanoparticles (NPs) can enter urine through glomerular filtration or renal tubular secretions. Particle size has been considered to be a key factor in determining renal clearance of NPs. NPs smaller than 10 nm can be rapidly and efficiently excreted into urine by glomerular filtration devices, while larger NPs can be blocked [22]. However, Su’s et al. studies have shown that QDs with hydrodynamic diameter less than 4.5 nm could not be completely excreted from the body, and were increasingly absorbed by the kidney during long-time blood circulation (up to 80 days) after intravenous injection [23]. This may suggest that particle size is not the only factor which determines the renal clearance of QDs. The clearance of QDs in the kidney may also be related to their surface charge, surface modification and other physical and chemical factors. At the same time, the long-term accumulation of QDs in the kidney may also have potential impact on kidney function.

Similar to most NPs, QDs will accumulate in the kidney if they are unable to pass through the glomerular filtration system during blood circulation. However, detailed information on the distribution of In-based QDs in the kidney in vivo, their effects on renal function and the underlying mechanisms is still limited. In this study, we investigated the renal toxicity of InP/ZnS QDs with different surface modifications (hydroxy, amino and carboxyl) in BALB/c mice. InP/ZnS QDs were prepared and three common ligands were functionalized on the surface of QDs—hydroxylated InP/ZnS QDs (hQDs), amino InP/ZnS QDs (aQDs) and carboxylic InP/ZnS QDs (cQDs), respectively. BALB/c mice were administered the three InP/ZnS QDs at the dose of 2.5 mg/kg body weight (BW) or 25 mg/kg BW through tail vein injection. The dietary patterns and body weight changes were recorded daily. Blood biochemistry, biodistribution and histopathological changes in the kidney were monitored at 1, 3, 7, 14 and 28 days after QDs exposure. Furthermore, we also detected inflammatory responses, oxidative stress markers and apoptosis pathway markers in kidney tissues to explore the mechanisms of QDs with different surface modifications in the kidney. We hope that our study can provide a reference for the biosafety evaluation of InP/ZnS QD.

## 2. Results

### 2.1. Characterization of InP/ZnS QDs

**Figure 1:**
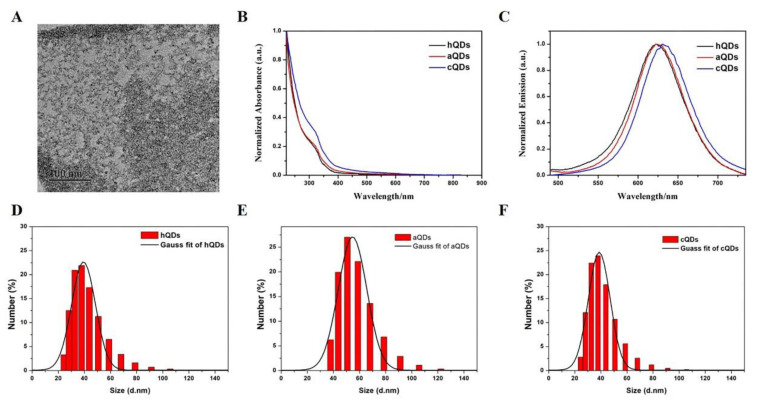
Characterization of three InP/ZnS QDs with different surface modifications. (**A**) TEM image of InP/ZnS QDs dispersed in toluene. (**B**) Normalized absorption spectra of hQDs, aQDs and cQDs. (**C**) Normalized PL spectra of hQDs, aQDs and cQDs recorded at room temperature in a 1 cm quartz cuvette (λex = 380 nm). (**D**) The hydrodynamic diameter of hQDs dispersed in deionized water. (**E**) The hydrodynamic diameter of aQDs dispersed in deionized water. (**F**) The hydrodynamic diameter of cQDs dispersed in deionized water.

InP/ZnS QDs before surface modification were steadily dispersed in toluene and the TEM image is shown in Figure 1A. The particle size of the QDs was about 5–8 nm. Then, the QDs were transferred from the organic phase to aqueous solution by commonly used ligands to obtain different functional groups (-OH, -NH_2_, -COOH) on the surface of the QDs. The hydrodynamic diameter and optical properties of three water-soluble InP/ZnS QDs were also investigated and the results are shown in Figure 1B–F. The hydrodynamic diameters of InP/ZnS-OH QDs (hQDs), InP/ZnS-NH_2_ QDs (aQDs) and InP/ZnS-COOH QDs (cQDs) were 50.89 ± 4.95 nm, 48.18 ± 1.77 nm and 55.93 ± 1.11 nm, respectively. The Zeta potentials of the three water-soluble InP/ZnS QDs were −22.70 ± 1.41 mV, −22.87 ± 0.61 mV, and −21.07 ± 1.64 mV. The absorption spectra are shown in Figure 1B and the first exciton peak of the three InP/ZnS QDs was around 350 nm. The photoluminescence (PL) spectra are shown in Figure 1C. The emission spectra of the three water-soluble QDs were narrow and symmetrical, and the emission peak was about 625 ± 10 nm following excitation at 380 nm. The above results illustrated that the three water-soluble InP/ZnS QDs with different modifications have uniform size and good optical properties.

### 2.2. Body Weight and Organ Weight/BW Coefficients

**Figure 2:**
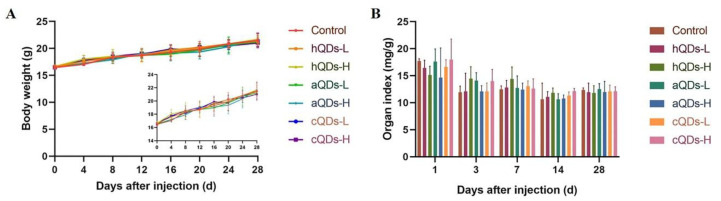
Body weight and kidney organ index in mice administered with 25 mg/kg BW QDs (H) or 2.5 mg/kg BW QDs (L). (**A**) The body weight curve of mice measured continuously for 28 days (Insert: magnified state of the body weight curve). (**B**) The kidney weight/BW coefficients of mice at different predetermined time points.

After intravenous injection of QDs in the tail vein, the behavior and mental status of mice were observed continuously, and the weight of mice was recorded daily. No mice died during the observation period. No significant changes were observed in food intake, fur, behavior and mental status after exposure of QDs. The body weight of mice was continuously monitored for 28 days and the data are shown in Figure 2A. All mice gained weight slowly but steadily. No significant differences were observed in body weight between the control group and the QDs-treated group over 28 days. After sacrificing the mice at the predetermined time points (1, 3, 7, 14 and 28 days), the kidneys were dissected, removed and weighed carefully. The kidney organ index was calculated as kidney weight (wet weight, mg)/BW (g). As shown in Figure 2B, there was no significant statistical difference in the kidney organ coefficient of mice between the QDs-treated groups and control group at the same sampling time.

### 2.3. Distribution of QDs in Kidney

**Figure 3:**
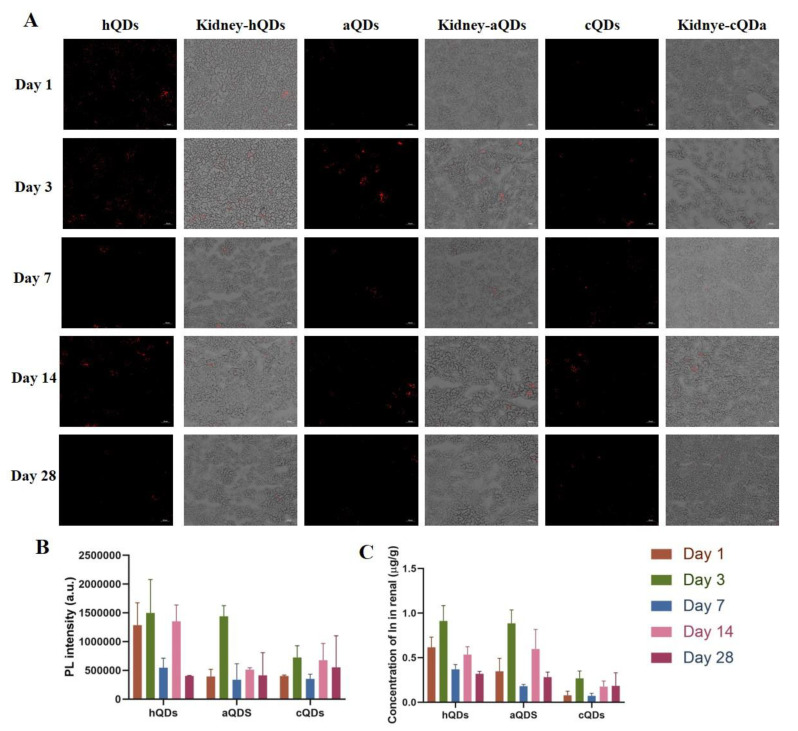
Distribution of QDs in kidney. (**A**) Representative fluorescence images of kidney tissue of mice administered with 25 mg/kg BW QDs at different predetermined time points (the red signal represented the fluorescence of QDs, scale bar: 50 μm). (**B**) Time-course of integrated mean PL intensity of QDs in renal tissue. (**C**) The In element concentration in kidney after administration of the three QDs at 25 mg/kg BW.

QDs could be driven through the whole body with the blood circulation, and may be distributed in the kidney and then cleared by the kidney. In order to study the distribution and clearance of QDs, fluorescence imaging and ICP-MS were used after mice were exposed to 25 mg/kg BW hQDs, aQDs and cQDs respectively. As showed in Figure 3A, a strong fluorescence of the QDs could be observed in the renal cortex of mice treated with QDs, including glomerulus and tubules. There was no obvious difference in the distribution region among the three kinds of QDs. No fluorescence was observed in the control group (data not shown). In order to quantitatively study the time-dependent changes in the PL intensity of QDs in the kidney, the average PL intensity of each image field was obtained from multiple slices of animals (*n* = 3). As shown in Figure 3B, the PL intensities of QDs in the kidney were dynamic and the fluorescence signals of the three InP/ZnS QDs were still observed up to the 28th day. The concentration of the In element was detected by ICP-MS to quantitate the accumulation of QDs in the kidney. The accumulation of the three QDs in the kidney reached the peak on Day 3 (hQDs: 0.91 ± 0.17 μg/g In; aQDs: 0.89 ± 0.15 μg/g In; cQDs: 0.27 ± 0.08 μg/g In), while the accumulation of cQDs in the kidney was less than that of the other two QDs. It should be noted that no In element was detected in the control group. The results showed that the three types of QDs could distribute in the kidney, and the accumulation of cQDs in the kidney was less than that of the other two QDs.

### 2.4. Changes of Serum Biochemical Parameters

**Figure 4:**
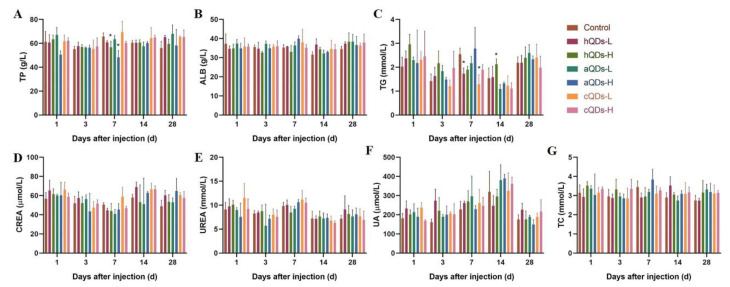
InP/ZnS QDs-induced changes in biochemical parameters of the serum of mice administered with 25 mg/kg BW QDs (H) or 2.5 mg/kg BW QDs (L). (**A**) TP level. (**B**) ALB level. (**C**) TG level. (**D**) CREA level. (**E**) UREA level. (**F**) UA level. (**G**) TC level. (* Significantly different compared to control group at the same sampling time, *p* < 0.05).

Serum biochemical indicators associated with renal function can reflect the injury effects of QDs on the kidney. Total protein (TP), Triglyceride (TG), albumin (ALB), creatinine (CREA), urea (UREA), uric acid (UA) and total cholesterol (TC)in the serum of mice at different sampling time points were measured and the results are presented in Figure 4 and Tables S1–S7. The TP levels in both the high dose hQDs group and high dose aQDs group were significantly lower than those in the control group on Day 7 (*p* < 0.05). The TG levels in both the low dose hQDs group and low dose cQDs group were significantly lower than those in the control group on Day 7 (*p* < 0.05). No significant differences in other indicators at different sampling time points were observed in QDs-treated groups when compared with the control group at the same sampling time. In addition, the TP, TG, CREA, UREA and UA levels in different QDs groups fluctuated on Day 3, 7 and 14 after treatment, and finally returned to the similar level to the control group. These results suggest that although different concentrations and surface modification of InP/ZnS QDs may lead to changes in some serum biochemical parameters, these changes are temporary and could eventually return to normal.

### 2.5. Histopathological Detection in Kidney Tissues

**Figure 5:**
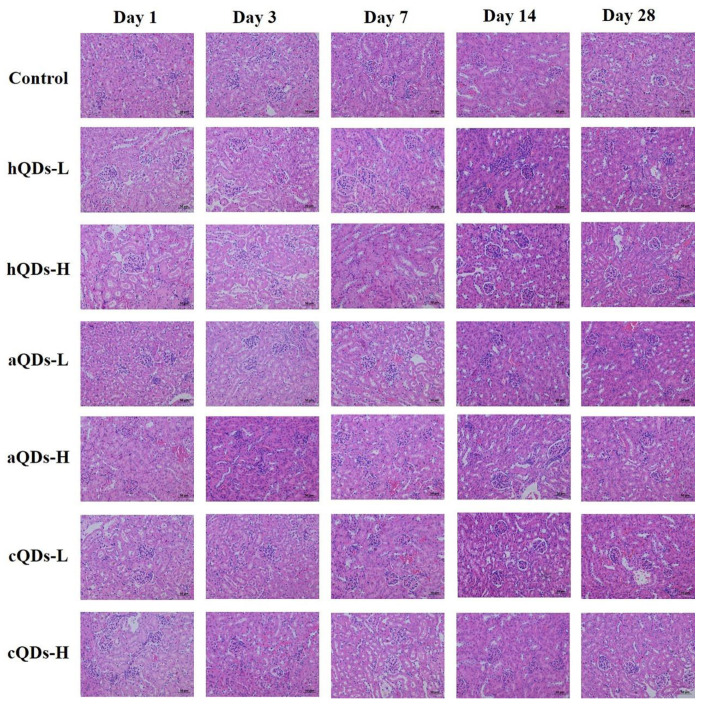
Representative histological images of kidney tissue from mice administered with 25 mg/kg BW QDs (H) or 2.5 mg/kg BW QDs (L) at different predetermined time points (scale bar: 50 μm).

Since all three water-soluble InP/ZnS QDs could accumulate in the kidney and the fluorescence of QDs can be detected up to 28 days, hematoxylin and eosin (H&E) staining was used to detect the histopathological changes in kidneys. The results are shown in Figure 5. No obvious histopathological changes were observed in the kidneys of all mice on Day 1, 3, 7, 14 and 28 after different concentrations and surface modifications of InP/ZnS QDs. Although the three different surface-modified InP/ZnS QDs can remain in the kidney for at least 28 days after tail vein injection, aQDs and hQDs in particular had more residues than cQD, and all of them did not cause significant histopathological changes in the kidney within 28 days.

### 2.6. Inflammatory Responses in Kidney Tissues

**Figure 6:**
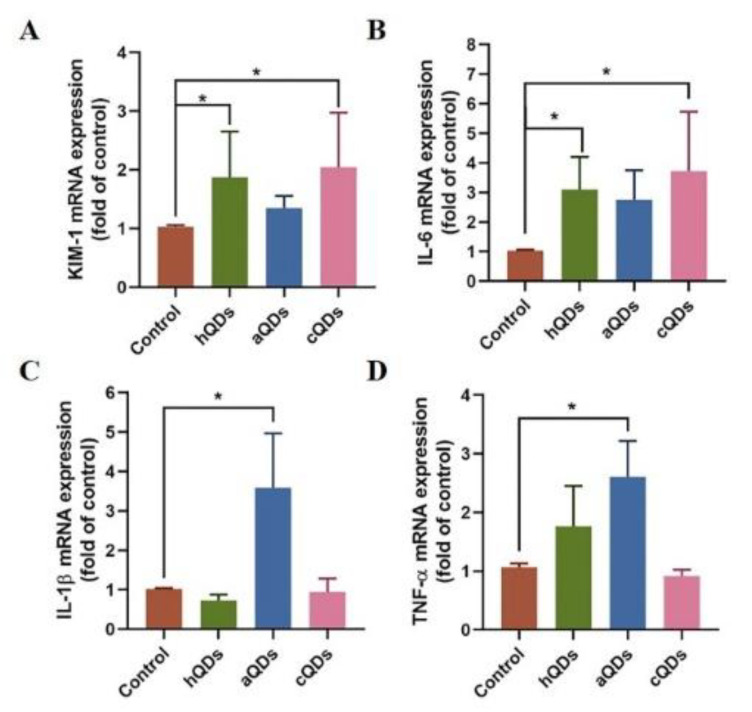
The mRNA levels of KIM-1 and inflammation-related genes in mouse kidney on Day 1 after administration of 25 mg/kg BW QDs. (**A**) KIM-1 mRNA levels. (**B**) IL-6 mRNA levels. (**C**) IL-1β mRNA levels. (**D**) TNF-α mRNA levels. (* Significantly different compared to control group, *p* < 0.05).

Kidney injury molecule-1 (KIM-1), a specific marker of renal injury, had a negligible expression level in normal renal tissues, but expressed rapidly and abundantly after renal ischemia or toxic injury. The mRNA expression of KIM-1 in renal tissues on Day 1 after high dose QDs exposure was detected and the results are shown in Figure 6A. The KIM-1 levels in the hQDs-exposed group and cQDs-exposed group were significantly higher than that in the control group (*p* < 0.05), indicating that high dose exposure of hQDs and cQDs could cause renal injury in mice. To further confirm the inflammatory response in the kidney, RT-PCR was used to detect the expression of IL-1β, IL-6 and TNF-α. As shown in Figure 6B–D, the IL-6 levels in both the hQDs and cQDs group were significantly higher than that in the control group (*p* < 0.05). The mRNA levels of IL-1β and TNF-α in the aQDs group were significantly higher than that in the control group (*p* < 0.05). The above results indicate that the three kinds of InP/ZnS QDs with different surface modifications could cause renal inflammatory responses and different degrees of renal injury.

### 2.7. Oxidative Stress Alterations in Kidney Tissues

**Figure 7:**
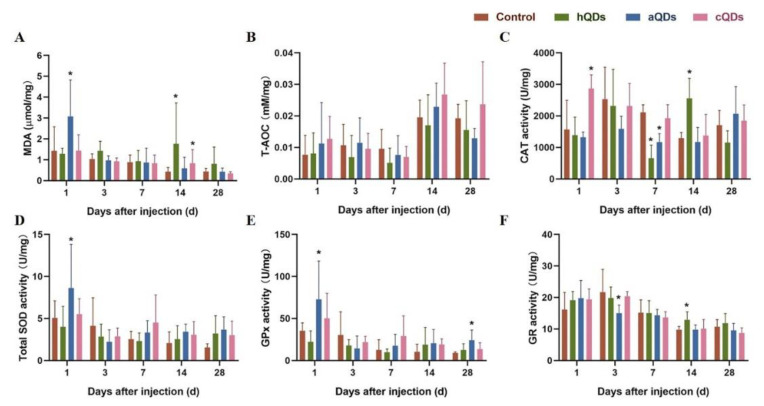
Oxidative stress levels in kidney tissues from QDs-treated mice at different predetermined time points after being administered with 25 mg/kg BW QDs. (**A**) MDA levels. (**B**) T-AOC levels. (**C**) CAT activities. (**D**) Total SOD activities. (**E**) GPx activities. (**F**) GR activities. (* Significantly different compared to control group, *p* < 0.05).

To investigate whether InP/ZnS QDs induced inflammatory responses and renal injury through oxidative stress, oxidative stress markers in renal tissues after high dose QDs exposure at different sampling time points were detected. The levels of malondialdehyde (MDA), total antioxidant capacity (T-AOC), catalase (CAT), total superoxide dismutase (SOD), glutathione peroxidase (GPx) and glutathione reductase (GR) are shown in Figure 7 and Tables S8–S13. The levels of MDA in the aQDs group on Day 1, and the hQDs and cQDs groups on Day 14 were significantly higher than in the control group (*p* < 0.05), which suggested that high dose InP/ZnS QDs exposure increased lipid peroxidation in kidney tissues after a single intravenous injection. However, the levels of MDA in the aQDs group and cQDs group on Day 7, 14 and 28 were significantly decreased when compared with MDA levels on Day 1. This suggested that QDs could cause oxidative damage to the renal tissue, but the oxidative damage weakened and returned to normal as time went on. The activities of T-AOC, CAT, total SOD, GPx and GR in the kidney all increased to different degrees within 28 days after the mice were treated with different surface modifications of InP/ZnS QDs. Increased levels of these antioxidant enzymes could improve the removal efficiency of reactive oxygen species (ROS) in kidney tissues, thereby alleviating the possible damage to kidney function caused by lipid peroxidation.

### 2.8. Apoptosis Pathway Alterations in Kidney Tissues

**Figure 8:**
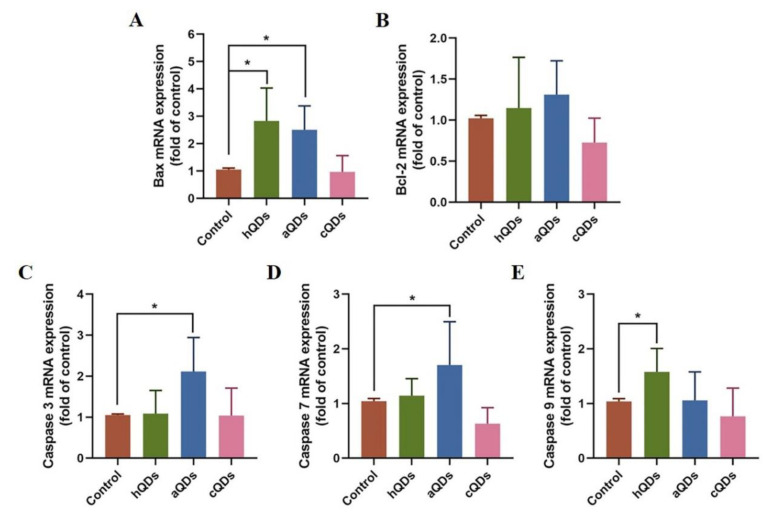
The mRNA levels of apoptosis-related genes in mouse kidney on Day 1 after being administered with 25 mg/kg BW QDs. (**A**) Bax mRNA levels. (**B**) Bcl-2 mRNA levels. (**C**) Caspase 3 mRNA levels. (**D**) Caspase 7 mRNA levels. (**E**) Caspase 9 mRNA levels. (* Significantly different compared to control group, *p* < 0.05).

On Day 1 after high dose QDs exposure, apoptosis-related genes in the kidney were detected and the results are shown in Figure 8. The expression levels of Bax were significantly up-regulated after exposure to high dose hQDs or aQDs, while Bcl-2 did not change. The expression levels of three key members of the Caspase family, Caspase 3, 7 and 9, were also detected. The Caspase 3 and 7 levels in the aQDs group were significantly up-regulated, and the Caspase 9 levels in the hQDs group were also significantly up-regulated. The expression levels of all the above genes in cQDs did not change. Taken together, these results suggest that hQDs and aQDs could promote apoptosis.

## 3. Discussion

The surface functionalization of QDs is one of the critical steps towards improving the physicochemical properties and biocompatibility of QDs [24]. However, the changes themselves may pose potential risks, which may endanger biological systems and lead to different toxicological patterns. Hoshino [25] reported that QD-COOH induced more cytotoxicity and DNA damage than QD-NH_2_ and QD-OH. They think this may be caused by the QD uptake in endosomes. Due to the binding of surface molecules to QDs via the electrical interaction between the sulfhydryl group and QD-covered zinc, surface molecules such as MUA may be separated and released into the cytoplasm under the acidic and oxidative conditions of endosomes. In our previous study [26], we found that cQDs and aQDs were able to enter the cells more easily than hQDs, while the cellular trafficking by QDs influenced their toxicity profiles. The nanoparticles that are more able to penetrate the cells will possibly result in higher cytotoxicity.

In this study, we investigated the renal toxicity of InP/ZnS QDs with different surface modifications (-OH, -NH_2_, -COOH) and different doses (2.5 mg/kg BW, 25 mg/kg BW) in mice after a single intravenous injection. The particle size of the three QDs was (5–6) nm. They were well dispersed in aqueous solution and had excellent optical properties. The kidney is one of the principal excretion pathways of NPs. It is generally believed that NPs with diameters less than 5.5 nm can be cleared through the kidney, while those with diameters larger than 15 nm could prevent renal excretion [27]. If NPs cannot be filtered by the glomeruli and deposited in the glomeruli after they have entered the body, they may cause damage to the kidney tissue and cause functional changes [28]. The distribution and accumulation of different types of QDs in the kidney were detected in this study. The strong fluorescence and In element concentration of the three QDs reached the peak on Day 3 after exposure, and all the three kinds of QDs could accumulate in the kidney for up to 28 days. However, the rate and concentration at which they accumulated in the kidney was not exactly the same, and the accumulation of cQDs in the kidney was less than that of the other two QDs. The observed fluorescence decreased gradually over the 28-day period. This may be due to the gradual decomposition or removal of QDs. Despite this, QDs cannot be completely removed within 28 days, and the fluorescence of QDs and In element could still be observed in kidney tissues on Day 28 after injection.

The accumulation of QDs in the kidney, and their degradation and breakdown over weeks may induce morphological and functional impairments in the kidney. In this study, we continuously monitored the weight changes and kidney organ coefficients of mice, and found that there were no statistical differences among the control group and QDs-treated groups. Furthermore, we examined the histopathological changes of mice. No obvious pathological changes in the kidneys were observed in the mice treated with different surface-modified QDs at different time points after intravenous injection. These results illustrate that In-based QDs did not cause significant histological changes in the kidneys, though they could accumulate in kidneys for a period of time. In-based QDs are recognized as environmentally friendly alternatives to Cd-based QDs. The results of a recent study on the toxicity of In-based QDs are similar to our results. Yaghini et al. [29] noted that In-based QDs could accumulate in the liver, spleen, lung and kidney for at least 90 days, but they did not observe organ damage or histopathological lesions in rats after 24 h, 1 week and 4 weeks following intravenous injection at 12.5 mg/kg BW or 50 mg/kg BW. In our previous research, studies of the long-term in vivo toxicity of PEGylated InP/ZnS QDs in BALB/c mice were carried out and no observable hematology, blood biochemistry, or organ histology changes were observed over an 84-day period after the mice were administered a very high dosage of InP/ZnS QDs [30].

For organisms, QDs belong to exogenous substances. When QDs are intravenously injected into the body, the body generates a large number of stress reactions, including changes in blood cells, blood biochemical indicators, inflammatory reactions, oxidative stress and so on. Serum protein and some unique enzymes can reflect the damage degree of kidney tissue and function. When the kidney undergoes inflammation or damage, these indicators will change significantly [31]. In the study by Tang et al. [32], higher levels of WBC, AST, ALT and CREA were observed two weeks after mice were injected with Cd-based QDs, which could reflect the kidney injury. CREA, UREA, UA, TP, ALB, TG and TC are important indicators of renal function. When these indicators change, it indicates that there may be inflammation of the kidney. In this study, biochemical parameters related to the kidney were measured after the mice were treated with different surface-modified QDs at different sampling time points. TP levels in both the high dose hQDs group and the high dose aQDs group were significantly lower than that in control group on Day 7. The TG levels in both the low dose hQDs group and the low dose cQDs group were significantly lower than that in the control group on Day 7. TP, TG, CREA, UREA and UA levels fluctuated on Day 3, 7 and 14 after treatment with different QDs, and finally returned to similar levels to the control group on Day 28. The results illustrated that the three kinds of InP/ZnS QDs with different surface modifications may lead to changes in renal function, and these changes are temporary and could eventually return to normal.

KIM-1, also known as T-cell Ig and mucin domain (TIM-1) or HAVCR1, is a type 1 transmembrane protein. As a specific marker of renal injury, it was barely expressed in normal renal tissues, but was massively induced after acute and chronic kidney injury [33]. Ji et al. [34] reported that KIM-1 expression levels were significantly increased after the mice were exposed to As or SO_2_, and the kidneys showed typical characteristics of diffuse sclerosing glomerulonephritis. In this study, although we did not observe significant histopathological changes, the KIM-1 levels were significantly up-regulated on Day 1 after exposure to hQDs and cQDs, which illustrated these QDs had already damaged the renal tissues. We further detected the expression level of inflammatory response-related genes in the renal tissues. The IL-6 levels in both the hQDs and cQDs groups were significantly higher than that in the control group, which was consistent with the results of KIM-1. The mRNA levels of IL-1β and TNF-α in the aQDs group were significantly higher than that in the control group. The above results indicate that the three kinds of InP/ZnS QDs could cause renal inflammatory response and renal injury.

Generally speaking, the damage caused by QDs included inflammatory reactions and oxidative stress. When nanomaterials enter the body, they could stimulate the production of ROS. In the physiological state, ROS are always in a dynamic balance between production and elimination, which is maintained by the antioxidant system in the body. If ROS cannot be removed normally and thus accumulate in the tissues, lipids in cells are oxidized to produce peroxides, which eventually leads to tissue damage and dysfunction [35,36]. MDA can directly reflect the severity of the free radical attack. CAT, SOD, GR and GPx are important defense lines of the antioxidant enzyme system. In this study, the levels of MDA in the aQDs group on Day 1, and in the hQDs and cQDs groups on Day 14, were significantly higher than those in the control group, which suggests that the three kinds of InP/ZnS QDs exposure increased the lipid peroxidation and ROS accumulation in kidney tissues after a single intravenous injection. However, the levels of MDA in the aQDs group and cQDs group on Day 7, 14 and 28 were significantly decreased when compared with the MDA levels on Day 1, which suggests that these QDs could cause oxidative damage to the renal tissue, but the oxidative damage eventually returned to normal. At the same time, almost all antioxidants, including T-AOC, CAT, total SOD, GPx and GR, increased to different degrees within 28 days. It was noted that the kidney was undergoing oxidative stress immediately after QDs exposure, and the body made a rapid defensive response through increasing the activities of SOD, CAT and other antioxidants to protect tissues and cells from oxidative damage. The above results suggest that three kinds of In-based QDs with different surface modifications can induce multi-level oxidative stress, break the balance of the oxidative/antioxidant system, and cause certain oxidative damages to the kidneys of mice. Then, the tissues can alleviate oxidative stress by increasing the activity of antioxidant enzymes, so as to restore the oxidative/antioxidant balance as much as possible and to protect its own function.

In addition, some studies pointed out that apoptosis was closely related to the tissue damage induced by QDs. Apoptosis could eliminate abnormal cells to keep the body healthy. The process of apoptosis is strictly controlled by multiple genes; among them, genes in the Bcl-2 family and Caspase family play an important role in the process of apoptosis. In this study, Bax expression levels were significantly up-regulated after exposure to high dose hQDs and aQDs, which indicates that hQDs and aQDs promote the process of cell apoptosis in kidneys. This conclusion is additionally supported by the increased expression of Caspase 3, 7 and 9 in the hQDs and aQDs groups. However, the expression level of all the above genes in cQDs did not change. Taken together, these results suggest that hQDs and aQDs exacerbated renal damage by promoting apoptosis and oxidative stress, while cQDs could cause renal damage mainly through oxidative stress.

## 4. Materials and Methods

### 4.1. Characterization of InP/ZnS QDs

Three kinds of InP/ZnS QDs—hydroxylated InP/ZnS QDs (hQDs), amino InP/ZnS QDs (aQDs), and carboxylic InP/ZnS QDs (hQDs)—were purchased from Najing tech Company, China. The size and morphology of the three QDs were characterized by transmission electron microscopy (TEM) (HT7700, HITACHI, Hitachi Japan). The hydrodynamic size distribution and zeta potential of the three QDs were characterized by zeta potential and particle size analyzer (Brookhaven Instruments Inc., Holtsville, NY, USA). Fluorescence spectra of the QDs were measured by fluorescence spectrophotometer (F-4600, HITACHI, Hitachi, Japan) and absorption spectra were measured by UV/Vis spectrophotometer (DU720, Beckman Coulter Inc., Breya, CA, USA).

### 4.2. Animals

Healthy female BALB/c mice aged six weeks were purchased from the Medical Laboratory Animal Center of Guangdong Province. The mice were housed in independent ventilation cages in a temperature-controlled and standardized sterile animal room with a 12-h day/night cycle at Shenzhen University. The mice were allowed to acclimate to the animal room for 7 days before the experiment began. Distilled water and sterilized food were available ad libitum. The animal study protocols were conducted in accordance with the Guide for the Care and Use of Laboratory Animals Center of Shenzhen University and approved by the Experimental Animal Ethics Committee of Shenzhen University (Permit No.2017002, 24 March 2017).

### 4.3. Animal Treatment and Sample Collection

Mice were randomly divided into seven groups: (1) hQDs high dose group (hQDs-H), treated with 25 mg/kg BW hQDs; (2) hQDs low dose group (hQDs-L), treated with 2.5 mg/kg BW hQDs; (3) aQDs high dose group (aQDs-H), treated with 25 mg/kg BW aQDs; (4) aQDs low dose group (aQDs-L), treated with 2.5 mg/kg BW aQDs; (5) cQDs high dose group (cQDs-H), treated with 25 mg/kg BW cQDs; (6) cQDs low dose group (cQDs-L), treated with 2.5 mg/kg BW cQDs; (7) control group, treated with physiological saline. The QDs were diluted in physiological saline and a single injection of QDs was administered through the tail vein with the volume of 100 μL per mouse. Mice in the control group were treated with the same volume of physiological saline through the same exposure pattern. The survival, food intake, fur, behavior, mental status and body weight of mice were observed and recorded carefully every day after exposure.

We define the day of tail vein injection as day 0, and at predetermined time points (1, 3, 7, 14 and 28 days) after treatment, six mice from each group were anesthetized with isoflurane. Blood samples were harvested from the posterior orbital venous plexus of the mice, gathered into procoagulant tubes, and used for blood biochemical analysis immediately. Then the mice were sacrificed by cervical dislocation and the kidneys were collected. Some pieces were fixed in tissue fixative for subsequent evaluation of histopathological changes. Other tissue samples were stored at –80 °C for detecting QDs distribution in the kidney, and oxidative stress markers.

### 4.4. Detection and Determination of QDs Distribution in Kidney

The mice were sacrificed on day 1, 3, 7, 14 and 28 after QDs exposure. A part of fresh kidney tissue was embedded in optimal cutting temperature compound (OCT) immediately and frozen in –80 °C for frozen section and fluorescence imaging. The tissue frozen section was processed by freezing microtome (CM3050S, Leica, Weztlar, Germany). The distribution of QDs in the kidney was observed by fluorescence microscopy (Axio Observer, ZEISS, Oberkochen, Germany). Another part of fresh kidney tissue was digested in the microwave digestion instrument and the concentration of In in the tissues was determined by inductively coupled plasma mass spectrometry (ICP-MS, PerkinElmer, Wellesley, MA, USA). The concentration of In in renal tissues was calculated with the following equations: [In] treated tissue (μg/g wet tissue) = [In] tissue suspension/wet weight of renal tissue.

### 4.5. Serum Biochemical Analysis

The blood of mice was collected and placed in disposable venous blood vessels containing separating gel. Serum was collected by centrifugation at 4 °C, 3500 rpm/min, for 15 min. The serum biochemical parameters were detected by automatic blood biochemical analyzer (BS-220, Mindray medical international limited, Shenzhen, China). All matching reagents were purchased from Mindray medical international limited, China. The specific biochemical indexes in this study included total protein (TP), Triglyceride (TG), albumin (ALB), creatinine (CREA), urea (UREA), uric acid (UA) and total cholesterol (TC).

### 4.6. Histopathological Examination

A portion of fresh kidney tissue was fixed in tissue fixative for histopathological examination. Fixed tissues were dehydrated in gradients with different concentrations of alcohol in an automatic tissue dehydrator (APS300S, Leica, Weztlar, Germany), and then were embedded in paraffin blocks by the paraffin embedding station (Leica, Weztlar, Germany). Tissues with 5 μm thickness were prepared by an ultra-thin semiautomatic microtome (RM2236, Leica, Weztlar, Germany). After staining with H&E, histopathological morphology was evaluated under the microscope (Axio Observer, ZEISS, Oberkochen, Germany) by an independent pathologist unaware of the treatment.

### 4.7. Oxidative Stress Markers Detection

After adding the appropriate amount of tissue lysate to the kidney tissues, tissues were cut up as fast as possible in an ice bath and homogenized with a tissue lyser (Shanghaijingxin Experimental Technology, Shanghai, China). The supernatant was collected by centrifuging at 4 °C, 10,000× *g*, for 10 min. After quantifying the protein in the supernatant, the levels of malondialdehyde (MDA), total antioxidant capacity (T-AOC), catalase (CAT), total superoxide dismutase (SOD), glutathione peroxidase (GPx) and glutathione reductase (GR) were estimated by the corresponding kits according to the manufacturer’s protocol. These kits were purchased from Beyotime Biotechnology co., ltd, Shanghai, China. Levels of MDA were determined by thiobarbituril colorimetry, and the absorbance of red reaction products MDA-TBA was measured at 532 nm by a multifunctional microplate reader (Infinite M plex, Tecan, Männedorf, Switzerland). T-AOC was measured by 2, 2′-azino-bis(3-ethylbenzothiazoline-6-sulfonic acid) (ABTS) colorimetry. CAT activities were measured by a colorimetric assay and the absorbance of red reaction products was measured at 520 nm. SOD activity was determined by a color development reaction based on WST-8 and the absorbance of reaction products was measured at 450 nm. GR activities were measured at 412 nm using chromogenic substrates 5,5′-dithio-bis-[2-nitrobenzoic acid] (DTNB). GPx activities were measured at 340 nm using organic peroxide reagent (t-Bu-OOH). All the results were shown as (levels of QDs exposure group)/(levels of control group) × 100%.

### 4.8. Quantitative Real-Time PCR

The total RNA was extracted from the renal tissues and was reverse transcribed with a commercial kit (GoScript™ Reverse Transcription Mix, Promega Corporation, Madison, WI, USA). Quantitative real-time PCR was performed with GoTaq^®^ qPCR Master Mix (Promega Corporation, Madison, WI, USA) on an Real-Time PCR system (TOptical Thermocycler^®^, Analytik Jena AG, Jena, Germany)). GAPDH mRNA amplified from the samples served as an internal control. The relative expression of each targeted gene was analyzed by the method of 2^−ΔΔCt^. The specific primers used for the gene expression analysis are shown in Table 1.

### 4.9. Statistical Analysis

Data were expressed as mean ± standard deviation (SD). All statistical analysis was performed with the SPSS 22.0 statistical software (International Business Machines Corporation, Amonk City, NY, USA) package and figures were drawn with the GraphPad Prism software package (Graphpad software, San Diego, CA, USA). The difference among the three groups was analyzed by one-way ANOVA. The differences were considered statistically significant if *p* < 0.05.

## 5. Conclusions

In summary, the renal toxicity of water-soluble InP/ZnS QDs with different surface functionalizations (-OH, -NH_2_, and -COOH) in BALB/c mice was investigated. Our results collectively indicated that different chemical surface modifications resulted in different biological behavior with different molecular mechanisms in vivo, which need to be taken into consideration in practical applications. Our study also indicated that the toxicity of QDs can be reduced by changing the surface modification, so as to make QDs more suitable for biomedical applications.

## Figures and Tables

**Table 1:** Primer sequences used for quantitative real-time PCR analysis.

Genes	Forward primers (5′-3′)	Reverse primers (5′-3′)
*Kim-1*	ACATATCGTGGAATCACAACGAC	ACAAGCAGAAGATGGGCATTG
*IL-1β*	GCAACTGTTCCTGAACTCAACT	ATCTTTTGGGGTCCGTCAACT
*IL-6*	TAGTCCTTCCTACCCCAATTTCC	TTGGTCCTTAGCCACTCCTTC
*TNF-* *α*	CCCTCACACTCAGATCATCTTCT	GCTACGACGTGGGCTACAG
*Caspase 3*	ATGGAGAACAACAAAACCTCAGT	TTGCTCCCATGTATGGTCTTTAC
*Caspase 7*	AAGACGGAGTTGACGCCAAG	CCGCAGAGGCATTTCTCTTC
*Caspase 9*	TCCTGGTACATCGAGACCTTG	AAGTCCCTTTCGCAGAAACAG
*Bax*	TGAAGACAGGGGCCTTTTTG	AATTCGCCGGAGACACTCG
*Bcl-2*	ATGCCTTTGTGGAACTATATGGC	GGTATGCACCCAGAGTGATGC
GAPDH	AGGTCGGTGTGAACGGATTTG	TGTAGACCATGTAGTTGAGGTCA

## Supplementary Materials

Supplementary Materials can be found at https://www.mdpi.com/1422-0067/21/19/7137/s1.

## Abbreviations

QDs	quantum dots
hQDs	InP/ZnS-OH QDs
aQDs	InP/ZnS-NH_2_ QDs
cQDs	InP/ZnS-COOH QDs
InP	indium phosphide
NPs	nanoparticles
TEM	transmission electron microscopy
OCT	optimal cutting temperature compound
TP	total protein
TG	triglyceride
ALB	albumin
CREA	creatinine
UA	uric acid
TC	total cholesterol
H&E	hematoxylin and eosin
MDA	malondialdehyde
T-AOC	total antioxidant capacity
CAT	catalase
SOD	total superoxide dismutase
GPx	glutathione peroxidase
GR	glutathione reductase
