# Supplementary material for: Nephrotoxicity Evaluation of Indium Phosphide Quantum Dots with Different Surface Modifications in BALB/c Mice
Source: Int J Mol Sci. 2020 Sep 27;21(19):7137. doi: 10.3390/ijms21197137 (PMC7582660; doi:10.3390/ijms21197137)
Supplement: Supplementary file 1 [file ijms-21-07137-s001.pdf]

Supplementary Materials

Nephrotoxicity evaluation of indium phosphide quantum dots with different surface modifications in BALB/c mice

Li Li, Tingting Chen, Zhiwen Yang, Yajing Chen, Dongmeng Liu, Huiyu Xiao, Maixian Liu, Kan Liu, Jiangyao Xu, Shikang Liu, Xiaomei Wang, Guimiao Lin and Gaixia Xu

Table S1 TP levels changes in serum of mice administered with 25mg/kg BW QDs (H) or 2.5mg/kg BW QDs (L) ($\bar{x}$ ± s, g/L).

	Day 1	Day 3	Day 7	Day 14	Day 28
Control	61.23 ± 8.58	55.17 ± 2.38	65.67 ± 3.03	60.47 ± 1.91	56.00 ± 5.73
hQDs-L	60.67 ± 6.52	57.60 ± 3.35	60.80 ± 1.42	60.40 ± 2.38	65.13 ± 1.55
hQDs-H	63.40 ± 3.42	56.90 ± 1.97#	56.70 ± 4.99*, #	60.67 ± 2.32	59.50 ± 4.00
aQDs-L	67.07 ± 6.20	56.37 ± 0.64#	64.30 ± 3.05	57.57 ± 3.52#	67.97 ± 7.39
aQDs-H	50.47 ± 3.26*	56.37 ± 2.15	48.30 ± 5.65*	60.20 ± 1.41	58.17 ± 13.46
cQDs-L	61.77 ± 5.10	55.37 ± 7.41	69.40 ± 8.88	64.37 ± 8.84	65.43 ± 0.90
cQDs-H	62.17 ± 2.39	57.35 ± 7.30	60.33 ± 1.40	64.83 ± 2.05	65.27 ± 5.85

*Significantly different compared to control group at the same sampling time, $P < 0.05$;
#Significantly different compared to Day 1 in the same treated group, $P < 0.05$.

Table S2 ALB levels changes in serum of mice administered with 25mg/kg BW QDs (H) or 2.5mg/kg BW QDs (L) ($\bar{x}$ ± s, g/L).

	Day 1	Day 3	Day 7	Day 14	Day 28
Control	37.27 ± 5.10	35.53 ± 1.12	35.27 ± 1.51	31.57 ± 1.45	34.53 ± 1.40
hQDs-L	34.63 ± 1.47	34.67 ± 1.94	35.80 ± 0.17	36.83 ± 2.99	37.27 ± 1.10
hQDs-H	34.83 ± 2.20	32.70 ± 0.35	33.00 ± 3.40	34.33 ± 1.15	38.37 ± 4.62
aQDs-L	37.36 ± 2.14	37.27 ± 0.90	36.33 ± 1.91	32.16 ± 1.82	38.37 ± 3.76
aQDs-H	34.83 ± 2.66	34.90 ± 1.08	39.97 ± 1.50	32.97 ± 0.45	36.60 ± 4.52
cQDs-L	35.87 ± 4.47	35.76 ± 0.66	37.97 ± 6.90	34.57 ± 4.50	36.30 ± 1.40
cQDs-H	35.93 ± 1.21	35.97 ± 1.63	35.20 ± 1.73	34.37 ± 2.50	37.90 ± 2.60

Table S3 TG levels changes in serum of mice administered with 25mg/kg BW QDs (H) or 2.5mg/kg BW QDs (L) ($\bar{x}$ ± s, mmol/L).

	Day 1	Day 3	Day 7	Day 14	Day 28
Control	2.03 ± 0.39	1.42 ± 0.30#	2.54 ± 0.26	1.55 ± 0.44	2.19 ± 0.25
hQDs-L	2.37 ± 0.31	1.63 ± 0.37#	1.72 ± 0.24*#	1.58 ± 0.45#	2.19 ± 0.31
hQDs-H	2.96 ± 0.42	2.16 ± 0.52#	1.90 ± 0.13#	2.13 ± 0.22*#	2.40 ± 0.45
aQDs-L	2.29 ± 0.14	1.84 ± 0.25	2.17 ± 0.29	1.09 ± 0.19#	2.60 ± 0.35
aQDs-H	2.17 ± 1.37	1.48 ± 0.10	2.78 ± 0.89	1.32 ± 0.06	2.33 ± 0.14
cQDs-L	2.31 ± 0.34	1.20 ± 0.26#	1.28 ± 0.41*#	1.22 ± 0.42#	2.40 ± 0.57
cQDs-H	2.46 ± 1.05	1.97 ± 0.69	1.89 ± 0.21	1.11 ± 0.23#	1.98 ± 0.48

*Significantly different compared to control group at the same sampling time, $P < 0.05$;
#Significantly different compared to Day 1 in the same treated group, $P < 0.05$.

Table S4 CREA levels changes in serum of mice administered with 25mg/kg BW QDs (H) or 2.5mg/kg BW QDs (L) ($\bar{x}$ ± s, μmol/L).

	Day 1	Day 3	Day 7	Day 14	Day 28
Control	56.67 ± 6.49	51.94 ± 7.40	50.50 ± 2.15	58.00 ± 3.33	48.83 ± 6.20
hQDs-L	65.33 ± 10.71	57.46 ± 6.31	44.57 ± 2.20#	68.87 ± 5.17	60.17 ± 5.23
hQDs-H	61.57 ± 5.44	52.20 ± 6.52	43.87 ± 7.81	51.13 ± 17.98	53.50 ± 7.45
aQDs-L	60.00 ± 1.56	56.40 ± 4.29	40.67 ± 4.88	51.06 ± 21.17	52.93 ± 4.66
aQDs-H	60.30 ± 13.45	43.37 ± 18.94	45.33 ± 6.35	62.83 ± 1.27	64.87 ± 12.97

cQDs-L	66.13 ± 7.71	47.20 ± 6.56#	58.90 ± 9.62	66.53 ± 6.54	60.50 ± 2.62
cQDs-H	58.80 ± 3.85	51.53 ± 4.08	46.97 ± 3.06#	66.63 ± 3.50#	57.63 ± 6.47

*Significantly different compared to control group at the same sampling time, $P < 0.05$;
#Significantly different compared to Day 1 in the same treated group, $P < 0.05$.

Table S5 UREA levels changes in serum of mice administered with 25mg/kg BW QDs (H) or 2.5mg/kg BW QDs (L) ($\overline{x}$ ± s, μmol/L).

	Day 1	Day 3	Day 7	Day 14	Day 28
Control	9.10 ± 1.46	8.25 ± 0.63	9.82 ± 0.74	7.21 ± 1.52	7.17 ± 0.68
hQDs-L	9.75 ± 1.59	8.46 ± 0.34	10.06 ± 1.03	7.14 ± 0.65	9.10 ± 2.84
hQDs-H	10.11 ± 0.89	8.73 ± 1.30	8.45 ± 1.28	7.62 ± 1.14#	8.18 ± 1.73
aQDs-L	8.97 ± 0.51	5.71 ± 4.47	9.25 ± 0.54	7.20 ± 1.17	7.63 ± 1.40
aQDs-H	7.53 ± 2.89	7.14 ± 0.75	10.60 ± 0.55#	7.35 ± 0.83	8.08 ± 1.32
cQDs-L	11.54 ± 2.99	7.99 ± 1.22#	11.14 ± 1.93	6.77 ± 0.94#	7.64 ± 1.63#
cQDs-H	9.22 ± 2.07	7.54 ± 1.43	10.48 ± 1.01	6.30 ± 0.43#	6.91 ± 1.85

#Significantly different compared to Day 1 in the same treated group, $P < 0.05$.

Table S6 UA levels changes in serum of mice administered with 25mg/kg BW QDs (H) or 2.5mg/kg BW QDs (L) ($\overline{x}$ ± s, μmol/L).

	Day 1	Day 3	Day 7	Day 14	Day 28
Control	181.57 ± 24.22	160.93 ± 16.25	228.10 ± 41.94	319.63 ± 107.11#	175.37 ± 23.87
hQDs-L	232.73 ± 39.21	273.57 ± 60.28	260.90 ± 10.66	246.72 ± 52.86	226.31 ± 33.04
hQDs-H	201.03 ± 24.10	220.67 ± 69.12	269.77 ± 56.05	295.13 ± 39.08#	175.66 ± 43.24
aQDs-L	213.17 ± 43.72	189.20 ± 11.25	296.56 ± 105.80	380.16 ± 80.37#	189.05 ± 8.35
aQDs-H	189.23 ± 45.11	200.1 ± 46.14	228.80 ± 19.41	389.60 ± 21.25#	149.07 ± 24.95
cQDs-L	237.70 ± 24.81	206.47 ± 10.01	261.03 ± 69.86	324.97 ± 93.82#	189.06 ± 18.25
cQDs-H	168.17 ± 6.60	202.37 ± 54.93	246.57 ± 45.02	361.00 ± 34.88#	216.40 ± 61.95

#Significantly different compared to Day 1 in the same treated group, $P < 0.05$.

Table S7 TC levels changes in serum of mice administered with 25mg/kg BW QDs (H) or 2.5mg/kg BW QDs (L) ($\overline{x}$ ± s, mmol/L).

	Day 1	Day 3	Day 7	Day 14	Day 28
Control	3.14 ± 0.41	2.95 ± 0.37	3.44 ± 0.31	2.90 ± 0.25	2.74 ± 0.26
hQDs-L	2.93 ± 0.42	2.87 ± 0.21	2.90 ± 0.23	3.52 ± 0.46#	2.74 ± 0.13
hQDs-H	3.52 ± 0.21	3.32 ± 0.53	2.95 ± 0.47	3.04 ± 0.11	3.16 ± 0.63
aQDs-L	3.36 ± 0.16	2.95 ± 0.17#	3.19 ± 0.14	2.74 ± 0.24#	3.32 ± 0.27
aQDs-H	3.03 ± 1.08	2.86 ± 0.22	3.83 ± 0.54	3.09 ± 0.17	3.19 ± 0.45
cQDs-L	3.15 ± 0.27	2.85 ± 0.53	3.09 ± 0.40	3.09 ± 0.59	3.10 ± 0.45
cQDs-H	3.46 ± 0.11	3.35 ± 0.50	3.27 ± 0.13	3.17 ± 0.28	3.15 ± 0.12

#Significantly different compared to Day 1 in the same treated group, $P < 0.05$.

Table S8 MDA levels changes in serum of mice administered with 25mg/kg BW QDs. ($\overline{x}$ ± s, μmol /mg)

	Day 1	Day 3	Day 7	Day 14	Day 28
Control	1.43 ± 1.14	1.03 ± 0.25	0.88 ± 0.34	0.43 ± 0.20#	0.44 ± 0.15#
hQDs-H	1.28 ± 0.27	1.42 ± 0.46	0.93 ± 0.51	1.76 ± 1.96*	0.81 ± 0.80
aQDs-H	3.07 ± 1.74*	0.97 ± 0.21#	0.86 ± 0.69#	0.59 ± 0.52#	0.43 ± 0.43#
cQDs-H	1.43 ± 0.77	0.92 ± 0.17	0.83 ± 0.39#	0.83 ± 0.66#	0.34 ± 0.34#

*Significantly different compared to control group at the same sampling time, $P < 0.05$;
#Significantly different compared to Day 1 in the same treated group, $P < 0.05$.

Table S9 T-AOC levels changes in serum of mice administered with 25mg/kg BW QDs ($\overline{x}$ ± s, mM/mg).

	Day 1	Day 3	Day 7	Day 14	Day 28
Control	0.0077 ± 0.006	0.0107 ± 0.006	0.0096 ± 0.006	0.0195 ± 0.005#	0.0192 ± 0.005#
hQDs-H	0.0081 ± 0.007	0.0069 ± 0.007	0.0051 ± 0.004	0.0170 ± 0.009	0.0155 ± 0.009

aQDs-H	0.0112 ± 0.013	0.0115 ± 0.0.008	0.0076 ± 0.006	0.0229 ± 0.007#	0.0129 ± 0.007
cQDs-H	0.0103 ± 0.007	0.0096 ± 0.005	0.0069 ± 0.003	0.0268 ± 0.009#	0.0237 ± 0.009#

#Significantly different compared to Day 1 in the same treated group, *P* < 0.05.

Table S10 CAT levels changes in serum of mice administered with 25mg/kg BW QDs ($\overline{x}$ ± s, U/mg).

	Day 1	Day 3	Day 7	Day 14	Day 28
Control	1569.78 ± 923.67	2530.44 ± 1015.88	2113.51 ± 238.18	1294.69 ± 181.72	1706.41 ± 463.75
hQDs-H	1386.69 ± 574.58	2318.44 ± 1159.73	660.76 ± 415.21*	2558.46 ± 632.99*, #	1154.94 ± 373.99
aQDs-H	1320.94 ± 167.12	1589.72 ± 401.77	1169.53 ± 365.33*	1172.69 ± 457.42	2069.37 ± 859.72#
cQDs-H	2868.23 ± 434.83*	2315.43 ± 712.46	1927.77 ± 425.88#	1376.30 ± 671.45#	1847.14 ± 500.10#

*Significantly different compared to control group at the same sampling time, *P* < 0.05;
#Significantly different compared to Day 1 in the same treated group, *P* < 0.05.

Table S11 SOD levels changes in serum of mice administered with 25mg/kg BW QDs ($\overline{x}$ ± s, U/mg)

	Day 1	Day 3	Day 7	Day 14	Day 28
Control	5.06 ± 2.04	4.13 ± 3.34	2.54 ± 0.92#	2.08 ± 1.32#	1.55 ± 0.44#
hQDs-H	4.01 ± 2.43	2.84 ± 1.48	2.32 ± 0.94	2.55 ± 1.59	3.20 ± 2.12
aQDs-H	8.62 ± 5.21*	2.23 ± 1.43#	3.35 ± 1.41#	3.44 ± 0.89#	3.67 ± 1.52#
cQDs-H	5.52 ± 1.83	2.86 ± 1.00#	4.50 ± 3.29	3.07 ± 1.57#	3.02 ± 1.66#

*Significantly different compared to control group at the same sampling time, *P* < 0.05;
#Significantly different compared to Day 1 in the same treated group, *P* < 0.05.

Table S12 GPx levels changes in serum of mice administered with 25mg/kg BW QDs ($\overline{x}$ ± s, U/mg)

	Day 1	Day 3	Day 7	Day 14	Day 28
Control	35.38 ± 9.31	30.40 ± 27.59	12.71 ± 12.02#	10.38 ± 9.09#	9.10 ± 1.23#
hQDs-H	22.33 ± 12.99	17.99 ± 6.87	9.97 ± 3.75	18.88 ± 20.58	12.53 ± 7.41
aQDs-H	72.85 ± 45.41*	14.38 ± 14.76#	17.52 ± 13.79#	20.54 ± 16.38#	24.23 ± 12.01*, #
cQDs-H	50.24 ± 29.66	21.92 ± 6.90#	29.21 ± 23.82	19.15 ± 6.51#	13.52 ± 7.64#

*Significantly different compared to control group at the same sampling time, *P* < 0.05;
#Significantly different compared to Day 1 in the same treated group, *P* < 0.05.

Table S13 GR levels changes in serum of mice administered with 25mg/kg BW QDs (H) ($\overline{x}$ ± s, U/mg)

	Day 1	Day 3	Day 7	Day 14	Day 28
Control	16.14 ± 5.47	21.69 ± 7.23	15.18 ± 4.06	9.76 ± 1.06#	10.71 ± 2.28
hQDs-H	19.14 ± 2.74	19.85 ± 3.45	15.06 ± 3.93#	12.93 ± 2.53*, #	11.87 ± 3.02#
aQDs-H	19.79 ± 5.58	15.01 ± 2.59*, #	14.35 ± 1.87#	9.77 ± 1.49#	9.57 ± 2.21#
cQDs-H	19.38 ± 3.33	20.37 ± 1.49	13.67 ± 1.82#	10.13 ± 2.88#	8.71 ± 1.65#

*Significantly different compared to control group at the same sampling time, *P* < 0.05;
#Significantly different compared to Day 1 in the same treated group, *P* < 0.05.
